# Partial Atomic Model of the Tailed Lactococcal Phage TP901-1 as Predicted by AlphaFold2: Revelations and Limitations

**DOI:** 10.3390/v15122440

**Published:** 2023-12-15

**Authors:** Jennifer Mahony, Adeline Goulet, Douwe van Sinderen, Christian Cambillau

**Affiliations:** 1 School of Microbiology & APC Microbiome Ireland, University College Cork, T12 K8AF Cork, Ireland; j.mahony@ucc.ie; 2 Laboratoire d’Ingénierie des Systèmes Macromoléculaires (LISM), Institut de Microbiologie, Bioénergies et Biotechnologie (IMM), Aix-Marseille Université—CNRS, UMR 7255, 13009 Marseille, France; adeline.goulet@univ-amu.fr

**Keywords:** bacteriophage, virion structure, *Lactococcus*, structural biology, AlphaFold2, P335, TP901-1

## Abstract

Bacteria are engaged in a constant battle against preying viruses, called bacteriophages (or phages). These remarkable nano-machines pack and store their genomes in a capsid and inject it into the cytoplasm of their bacterial prey following specific adhesion to the host cell surface. Tailed phages possessing dsDNA genomes are the most abundant phages in the bacterial virosphere, particularly those with long, non-contractile tails. All tailed phages possess a nano-device at their tail tip that specifically recognizes and adheres to a suitable host cell surface receptor, being proteinaceous and/or saccharidic. Adhesion devices of tailed phages infecting Gram-positive bacteria are highly diverse and, for the majority, remain poorly understood. Their long, flexible, multi-domain-encompassing tail limits experimental approaches to determine their complete structure. We have previously shown that the recently developed protein structure prediction program AlphaFold2 can overcome this limitation by predicting the structures of phage adhesion devices with confidence. Here, we extend this approach and employ AlphaFold2 to determine the structure of a complete phage, the lactococcal P335 phage TP901-1. Herein we report the structures of its capsid and neck, its extended tail, and the complete adhesion device, the baseplate, which was previously partially determined using X-ray crystallography.

## 1. Introduction

Bacteriophages are the most abundant biological entity on Earth with an estimated 10^31^ particles being present on our planet. Among these, tailed bacteriophages with double-stranded DNA genomes are the most dominantly observed and reported in the literature [1]. Until recently, tailed phages were classified within the Caudovirales order and described based on their morphology, possessing either a short (*Podoviridae*) or long tail that is contractile (*Myoviridae*) or non-contractile (*Siphoviridae*). Given the extent of their genetics, host species, and ecological diversity, the taxonomy of phages has recently been re-established to reflect these evolutionarily relevant factors and to reduce reliance on morphology-based assignments [2]. The current viral class Caudoviricetes encompasses 14 families assigned to one of four orders, while a further 33 families have been identified that have yet to be assigned to an order. Irrespective of the (sequence-based) taxonomy of phages, their morphology has a significant bearing on the types of interactions they will establish with their respective hosts [3,4].

**Figure 1:**
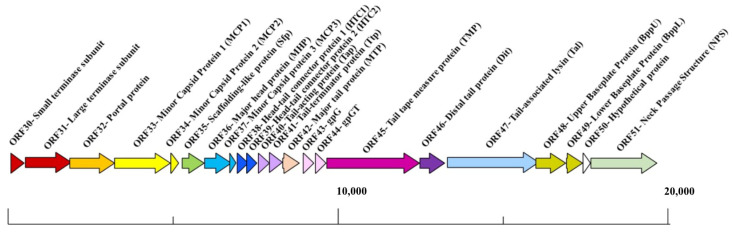
Schematic representation of the structural module of TP901-1. The functions are indicated above the arrows and the scale bar is presented at the base of the schematic, measured in base pairs (bp). This figure and associated functions are based on those described in reference [17].

Phages with long, non-contractile tails (formerly called the *Siphoviridae*) are the most abundant in nature and several of these have become models to study phage–host interactions, including the *Lactococcus* phages TP901-1 and p2, *Streptococcus thermophilus* phage STP1, *Bacillus subtilis* phage SPP1, and *Escherichia coli* phages lambda and T5 [5,6,7,8,9,10]. These phages are genetically distinct; however, in many cases, functional modules within their genomes are syntenic [11]. Furthermore, despite sequence disparity between phages of the same or distinct bacterial host species, the structure of their components is often conserved [4,12,13,14,15]. The temperate lactococcal P335 phage TP901-1 is particularly well characterized with respect to its three-dimensional structure and the interactions with its host [5,16,17,18]. Furthermore, it has been employed as a model to understand the phenomenon of lysogeny and the factors that underpin the lytic–lysogenic lifestyle decision process [19,20,21]. TP901-1 structural module encompassing 22 genes that are responsible for the biosynthesis of the phage head, tail, baseplate, and DNA packaging machinery (Figure 1).

TP901-1 possesses an isometric capsid and a long non-contractile tail of approximately 135 nm [22]. At the distal end of its tail, TP901-1 presents a large, multi-protein adhesion device, termed a baseplate, which is composed of the tail tape measure protein (TMP), distal tail protein (Dit), tail-associated lysin (Tal), and upper (BppU) and lower (BppL) baseplate proteins [5,23]. The BppL component is the bona fide receptor-binding protein (RBP) and presents as 18 trimers in the whole baseplate structure, which likely recognize and bind to cell wall polysaccharides present on the surface of the host lactococcal cell. Its exquisite host specificity is based on its heterologous expression of the *Lactococcus lactis* genetic region, encoding host-specific glycosyltransferases associated with cell wall polysaccharide biosynthesis [24].

The adhesion device of TP901-1 is in an infection-ready conformation and does not require activation by divalent cations, such as calcium, as is the case for certain tailed phages including the lactococcal phage p2 [25]. Mutational analyses of the TP901-1 gene coding for the capsid and tail proteins have provided insights into the assembly of the mature virion and the function of several previously uncharacterized gene products [17]. Furthermore, detailed genetic and structural analyses of the tail and baseplate proteins have rendered TP901-1 one of the best-characterized phages capable of infecting Gram-positive bacteria [5,22,26].

The recent development of AlphaFold2 (AF2) has significantly enhanced and advanced our capabilities to predict individual and multi-protein structures [27,28,29] and has transformed the field of structural biology. The ever-increasing number of phage genome and protein sequences and protein structures in public databases reinforces the need of reliable and rapid methods for the determination of (multi-component) protein structures and the associated protein functionality. In the present study, we present predicted structures of the TP901-1 capsid and neck, its extended tail, and its complete baseplate structure, which was previously partially determined with X-ray crystallography. AF2 reliably predicted the structures of large parts of the phage in most cases yet was unsuccessful in a small number of cases, in which chaperone-aided folding was probably the reason for such structure prediction failure.

## 2. Materials and Methods

We performed predictions using either a Colab notebook running AlphaFold v2.3.1 (https://colab.research.google.com/github/deepmind/alphafold/blob/main/notebooks/AlphaFold.ipynb, 1 January 2023) on Nvidia A100 40 Gb GPU, or HPC resources from GENCI-IDRIS running AlphaFold v2.3.1 on a Jean Zay supercomputer A100 80Gb GPU. The retry number was set to 20 and no relaxation was performed. Five models were generated for each prediction. Colab was used with small complexes while Jean Zay was used for large complexes requiring longer calculation times.

The virion structure of the tailed phage that we present here is one of the most complete to date. For such prediction-based structural work, the validity of the predicted structures is an important issue. AF2 has been recognized to provide high-quality structures provided that the pLDDT values are sufficiently high, i.e., above 80% [27,28,29,30]. Here, we checked the structure’s validity at three levels. First, internal to AF2, we only accepted structures that met the AF2 internal validation score given by the pLDDT values, i.e., when the pLDDT values were above 70% in the folded regions. Although loops often occur below this threshold, they do not significantly affect the overall fold. Moreover, we also examined the PAE plots, which give confidence scores of protein–protein interactions, and verified that the pLDDT values in the protein complex were at least equal or higher than those of the non-complexed structure. Second, we used an external validity criterion by assessing the agreement between the predicted structures and the experimental TP901-1 nsEM 3D reconstructions in the 15–25 Å-resolution range. We also used the available crystal structures to evaluate the capabilities and limitations of AF2’s predictions. As a third validation approach, we checked with the Dali server [31] whether the predicted folds had already been reported in the PDB. The plots of the pLDDT values, stored in the PDB file as B-factors, as well as the PAEs are shown in Supplementary Materials. Additionally, we used the PISA server [32] to analyze the quality of the interactions within our assemblies.

The pLDDT values of the predicted structures, stored in the PDB file as B-factors, as well as the PAEs were plotted and are shown in the Supplementary Materials, Figure S1. The final predicted protein or domain structures were submitted to the Dali server [31] to identify their closest structural homologs in the PDB. Visual representations of the structures were prepared with ChimeraX [33]. Coot [34,35] was used to assemble and visually analyze the predictions.

## 3. Results

### 3.1. The TP901-1 Capsid

**Figure 2:**
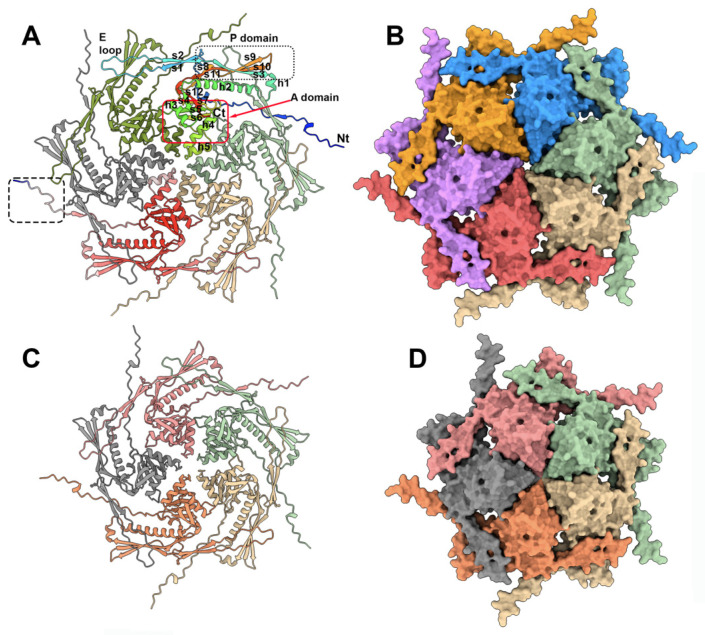
TP901-1 capsid’s hexon and penton structures. (**A**) Ribbon view of the predicted hexon structure. One monomer is rainbow colored and its domains are labeled. The monomer at the bottom of the figure is colored according to the pLDDT values (the quality of the prediction), from blue (low) to red (high). Note the low-confidence structure of the N-terminus (bottom dashed box). (**B**) Surface representation of the hexon (same orientation and scale as in (**A**)). (**C**) Ribbon view of the predicted penton structure. (**D**) Surface representation of the penton (same orientation and scale as in (**C**)).

Phage capsids are robust containers that carry and protect the viral genome packaged within its internal cavity [36]. Previously, the phage TP901-1 capsid structure has been determined via negative staining electron microscopy (nsEM) and single-particle analyses applying icosahedral symmetry at a 15 Å resolution [22]. The mature capsid (660 Å wide) assembles 60 hexamers (hexons) and 11 pentamers (pentons) of the ORF36 major capsid protein (MCP), with a T = 7 symmetry (EMD-2133). The 12th penton is replaced by a dodecamer of the portal protein. Here, we predicted the structures of these MCP hexamers and pentamers with confident scores, except for the N-terminus residues 1 to 15 (Figure 2, Supplementary Materials Figures S1 and S2). In both assemblies, the capsid MCP monomer contains the classical domains of a phage capsid protein, including, from the N-terminus to the C-terminus, the β-hairpin “E-loop”, the “P-domain” β-sheet, and a central “A-domain” formed of β-strands 4–6 and α-helices 3–5 (Figure 2A,C). While the A-domains form compact structures in the hexons and pentons, the looser E-loops cover an adjacent monomer (Figure 2B,D).

In the entire capsid, each penton is surrounded by five hexons. Therefore, in order to analyze the protein contacts at the hexon–hexon and hexon–penton interfaces, we attempted to predict the structures of two hexons and of one hexon with one penton by feeding AF2 with 12 and 11 MCP sequences, respectively. However, in both cases, AF2 returned the hexons and penton separated, thus limiting the possibility to examine the interfaces. In order to overcome this limitation and to evaluate the predictions in the context of the whole capsid, we fitted the two predicted hexons and one penton (in the absence of residues 1–15) in the capsid nsEM 3D reconstruction (cross-correlation values of 0.81 (hexon) and 0.78 (penton)) (Supplementary Materials, Figure S3). The TP901-1 capsid is structurally very similar to the coliphage HK97 [37], as reported by Dali (PDB ID: 1ohg; Z-score = 18.1; rmsd = 3.0 Å, for 248 aligned residues of the 280 residues in total).

### 3.2. The Procapsid Scaffolding Protein

**Figure 3:**
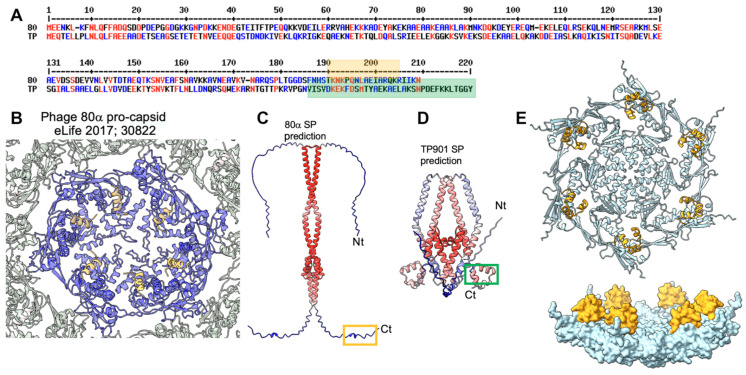
The procapsid’s scaffolding proteins (SPs). (**A**) Amino acid alignment between the scaffolding proteins of staphylococcal phage 80α and lactococcal phage TP901-1. (**B**) Ribbon representation of the cryoEM structure of a phage 80α MCP hexamer (blue) in complex with the C-terminus of the scaffolding protein (orange; see also the orange box in (**A**)) [1]. (**C**) AF2 prediction of the full-length phage 80α scaffolding protein. The orange box corresponds to the orange helix in (**B**). (**D**) AF2 prediction of the full-length phage TP901-1 scaffolding protein. The green-boxed helices correspond to the green box in (**A**). (**E**, **top**) Ribbon representation of a phage TP901-1 MCP hexamer (light blue) in complex with the C-terminus of the scaffolding protein (orange; see also the green box in (**A**)). (**E**, **bottom**) Surface view of the same structure rotated 90°.

Procapsids (virion precursors without DNA) are assembled from the dodecameric portal via the addition of MCP hexons and pentons [38]. This process is promoted by scaffolding proteins that have been proposed to establish bridges between capsid MCPs [39,40]. Little is known, however, about the structure of these scaffolding proteins, either alone or when they are attached to the MCPs. The procapsid structure of the *Staphylococcus aureus* phage 80α reveals the presence of an α-helix attached to each MCP on the internal face of the procapsid [39] (Figure 3A,B). This short α-helix, which belongs to the C-terminal end of the scaffolding protein, is only a small portion of the phage 80α’s full-length scaffolding protein (209 residues). The putative scaffolding protein of TP901-1 is slightly longer than that of 80α at its C-terminus (220 residues) (Figure 3B). Therefore, we predicted their structures, which assemble dimers, with good statistics (Figure 3C). However, while the 80α’s scaffolding protein shows an unstructured C-terminal end, that of TP901-1 shows three well-folded C-terminal α-helices (Figure 3D). We also predicted the structure of the complex between these C-terminal helices and an MCP hexon, and we found that each MCP is bound to this helical domain on the internal face of the hexon (Figure 3E,F). Scaffolding proteins (SPs) bind to the MCPs from the non-mature capsid and are released upon maturation [39]. Here, the MCP to which the SP binds resembles MCPs from mature capsids, indicating that AF2 is unable to discriminate between non-mature and mature MCPs for SP binding. 

### 3.3. Dodecameric Portal and Adaptor and the Hexameric Stopper

#### 3.3.1. The Dodecameric Portal (ORF32)

Phage portals are dodecameric assemblies that replace a penton in the capsid structure [41,42,43]. They are bound to a dodecameric adaptor, which is also connected to a hexameric stopper. The portal/adaptor/stopper complex is often referred to as the neck or the genome gatekeeper [44]. The TP901-1 portal dodecameric assembly comprises 5484 residues in total (12 × 457 residues), which is beyond the prediction capability of AF2, currently limited to ~4000 residues. Therefore, we used a multi-step approach to overcome this limit and obtain the structures of the portal dodecamer. First, we predicted the structure of a portal trimer to identify the regions of the portal protein that are not involved in self-assembly, essentially the N-terminal (1–64) and the C-terminal (380–457) ends, and define “short” versions of the portal protein (316 residues) and its dodecamer (3792 residues) for structure prediction. We confirmed that the predicted structures of the monomers in the “short” dodecamer superimposed perfectly onto that of the predicted full-length portal. Then, a full-length dodecameric portal was formed by superimposing the full-length monomers onto each subunit of the “short” dodecamer with Coot [35].

**Figure 4:**
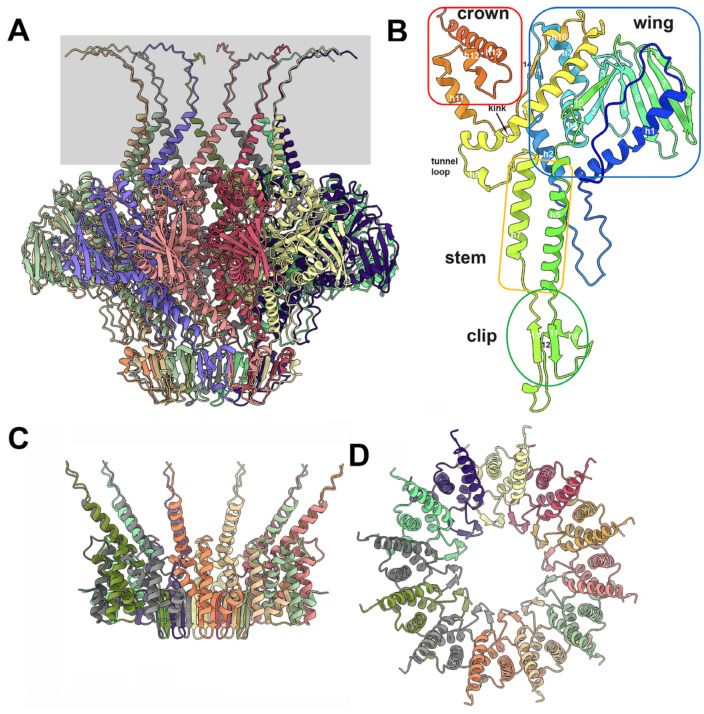
The structures of the dodecameric portal and adaptor. (**A**) The portal dodecamer has a size of 160 × 110 Å. The C-terminal segments exhibit low pLDDT values and have been boxed in gray. (**B**) The structure of the portal monomer with the different domains conserved in all portals. The C-terminal extension is poorly predicted and is disordered. (**C**) Lateral view of the adaptor with the extended C-terminal α-helices and the central β-sheet. (**D**) The same view rotated 90°, relative to (**C**).

**Figure 5:**
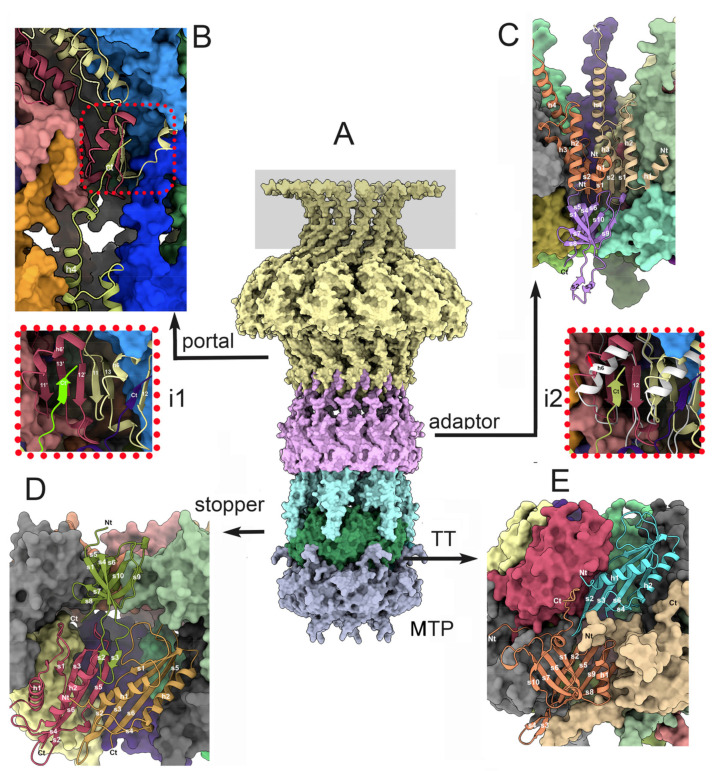
Protein structure from the portal to the major tail portein. (**A**) Dodecameric portal (yellow) and adaptor (violet), hexameric stopper (light blue), tail terminator (TT, green), and major tail protein (MTP, gray). The C-terminal segments exhibit low pLDDT values and have been boxed in gray. (**B**) The portal/adaptor interface (close-up view in inset one, **i1**) involves four b-strands: portal i, s13 and s11 (anti-parallel); portal i + 1, s12 and adaptor C-terminal b-strand stacks against a-helix 6; and portal i + 1, a-strands s11 and s13. (**C**) The adaptor/stopper interface involves two adaptors (beige and orange) and one stopper (violet). Contacts originate from the stopper’s N-terminus and loops joining the a-strands of the three monomers. The insertion of the adaptor displaces a-helix 6 (inset two, **i2**). (**D**) The stopper/TT interface: The long stopper’s b-hairpin loop s2–s3 (light green) is inserted between two TT monomers (red and beige). Other contacts involve the long TT’s loops h1–s1 and s3–s5. (**E**) The TT/MTP interface: The MTP i − 1 N-terminus (beige) is inserted between MTP i (orange) and TT (light blue), and comprises the majority of TT/MTP interactions. The MTP C-terminus is inserted vertically between two TT monomers.

A phage TP901-1 portal dodecamer has dimensions of ~160 Å (external diameter) and ~110 Å (height excluding the C-terminus), with a central channel of ~30Å in diameter (Figure 4A and Figure 5A,B). As with other phage portal proteins, each monomer contains domains named the wing, stem, clip, and crown domains [41,42,43,44] (Figure 4B). The wing is the central core of the portal. The C-terminal part of the crown exhibits very-low pLDDT values (Supplementary Materials, Figures S1 and S4), meaning that they are probably unstructured in the absence of DNA. 

The wing domains form the central core of the portal assembly. The wing starts with an extended stretch (residues 1–18) followed by a long α-helix (residues 20–45), a loop (residues 46–67), and a second α-helix (residues 68–80). The first β-strand is followed by two α-helices (residues 81–121) and a β-hairpin (residues 122–142). A β-sheet of six antiparallel β-strands (residues 144–201) is followed by a β-strand antiparallel to the β-hairpin and a loop connected to the first α-helix (residues 223–246) of the stem.

The clip domain follows with β-strand 11, a loop, α-helix 6, β-strand 12, another loop, and β-strand 13. β-strands 11 and 13 form an antiparallel β-sheet. The β-strand 13 is connected to the second α-helix of the stem (residues 292–312), which is followed by the tunnel loop and a long, kinked α-helix (residues 326–363) conserved in phage portal proteins. After a short α-helix, the last β-strand 14 closes the wing by forming an antiparallel β-structure with β-strand 1, which is followed by an extended stretch connected to the three α-helices of the crown (385–425). It is noteworthy that the C-terminal end of the portal protein (residues 420–457) is predicted with low confidence. Interactions between portal monomers are strong, as they involve 4100 Å^2^ of the buried surface area, which is ~14% of the total surface area (29,000 Å^2^). As mentioned above, these interactions involve most of the residues along the protein chain, with the exception of the N- and C-termini. The Dali server reported an impressively strong hit with the *B*. *subtilis* phage SPP1 portal protein (PDB ID: 3jes-U; Z-score = 27.4; rmsd = 2.6 Å; lali: 348/370; [41]) and a similarly good hit with the *E. coli* podophage T7 portal (PDB ID: 6qwp-F; Z-score = 20.7; rmsd = 5.5 Å; lali: 352/486; [45]).

#### 3.3.2. The Dodecameric Adaptor (ORF38)

The predicted structure of the adaptor protein starts with an α-helix (h1), followed by two additional a-helices (h2, h3), an anti-parallel β-hairpin (s1, s2), and a long C-terminal a-helix (h4) (Figure 4B,C and Figure 5C). In the predicted adaptor dodecameric assembly, the twelve b-hairpins form a cylindrical b-sheet of 24 antiparallel b-strands. This cylinder, which is the conduit for DNA passage, has a diameter of ~28 Å. The C-terminal a-helix h4 (residues 85–96) is followed by an unstructured segment (residues 86–105) and the C-terminal b-strand (residues 105–110). The interactions between adjacent adaptor monomers are strong, as they involve a buried surface area of 1320 Å^2^ of a total surface area of 8900 Å^2^ (~15%). These intermolecular interactions involve the b-barrel, as well as a-helix 3 sandwiched between a-helices 2 and 1 of an adjacent monomer, and a-helix 2 from adjacent monomers. Submission to the Dali server reported rather poor results for the bacteriophage T7 tail protein gp12 (PDB ID: 7ey7-T; Z-score = 6.5; rmsd = 4.7 Å; lali: 86/193) and the podophage T7 adaptor (PDB ID: 2kz4; Z-score = 9.1; rmsd = 3.3 Å; lali: 94/112), as well as the native GTA particle stopper (PDB ID: 7box-T; Z-score = 6.4; rmsd = 4.8 Å; lali: 88/195). Therefore, the structure of the adaptor protein reported here seems to be novel, in particular its 24-strand b-barrel.

#### 3.3.3. The Portal/Adaptor Complex

Since the prediction of a portal/adaptor dodecamer was also beyond the possibilities of AF2, we predicted the structure of a complex between 12 clip domains of the portal protein (residues 246–288) and 12 adaptor proteins, which are known to interact in portal/adaptor assemblies. This clip/adaptor dodecamer was superimposed onto the full-length portal dodecamer to obtain a complete portal/adaptor dodecamer.

The portal/adaptor interface involves a limited number of residues (Figure 5A,B). In particular, the C-terminal b-strand (residues 105–110) forms a four-strand b-sheet together with two adjacent portal monomers (Figure 5, inset one), leading to a very modest interaction surface area of 710 Å^2^ (~1.8% of the portal’s total surface area and ~7.8% of the adaptor’s total surface area). This limited interaction results in a poor compactness at the portal/adaptor interface, with “holes” in the adaptor’s structure. 

#### 3.3.4. The Adaptor/Stopper (ORF39) Complex

We predicted the structure of six stopper proteins together with twelve adaptor proteins (1938 residues in total) in a single-job submission. The adaptor (sometimes referred as the “connector” or “head-to-tail connector”) structure in this complex is similar to that observed in the adaptor/portal complex, with the exception of its C-terminus, which is predicted as an a-helix, with poor statistics in the absence of the clip domain (Figure 5C). Each stopper monomer contains a b-sandwich of two b-sheets, with four antiparallel b-strands each, a long b-hairpin, and an a-helix; b-strands 5, 6, 10, and 9 form the first b-sheet, and b-strands 4, 1, 7, and 8 form the second one. The b-hairpin (b-strands 2 and 3) is an extension of b-strands 1 and 4. A unique a-helix is found between b-strands 5 and 6. 

The central cavity of the stopper’s hexameric structure is partly obstructed by the six a-helices, while the b-hairpins are projected out of the central axis. The interactions between two adjacent stopper monomers involve the loops between the b-strands and the a-helix, as well as the a-helices themselves. Therefore, the interactions between adjacent monomers are weak, with a buried surface area of ~400 Å^2^ of the total 7580 Å^2^ surface area (5.2%). Consequently, AF2 was unable to predict the stopper hexameric structure alone, likely because of this weak surface area. The two best results obtained with the Dali server were those for the NMR structure of a *Shigella flexneri* protein, which likely comes from a temperate phage (PDB ID: 2kz4; Z-score = 9.1; rmsd = 3.3 Å; lali: 94/112; unpublished) and the native GTA particle stopper (PDB ID: 6te9; Z-score = 7.6; rmsd = 4.0 Å; lali: 84/110; [46]).

The adaptor–stopper interface is rather flat. Each stopper monomer interacts with three adaptor monomers with buried surface areas of 770 Å^2^, 300 Å^2^, and 260 Å^2^, reaching a total interface area of 1330 Å^2^ on a total surface area of 7580 Å^2^ (17.5%), a value indicating that the adaptor dodecamer and the stopper hexamer strongly interact with each other. (Figure 5A,D). Additional contacts involve the stopper loops s4–s5 and s6–s7 with the adaptor loops h1–h2, and the stopper loops s9–s10 with the adjacent adaptor loops h1–h2. 

#### 3.3.5. The Collar and Neck Passage Proteins

A low-resolution (20 Å) nsEM structure of the TP901-1 neck has been reported previously [22]. We used ChimeraX to fit the predicted portal/adaptor/stopper-associated atomic model in this 3D reconstruction (EMD-2227).

**Figure 6:**
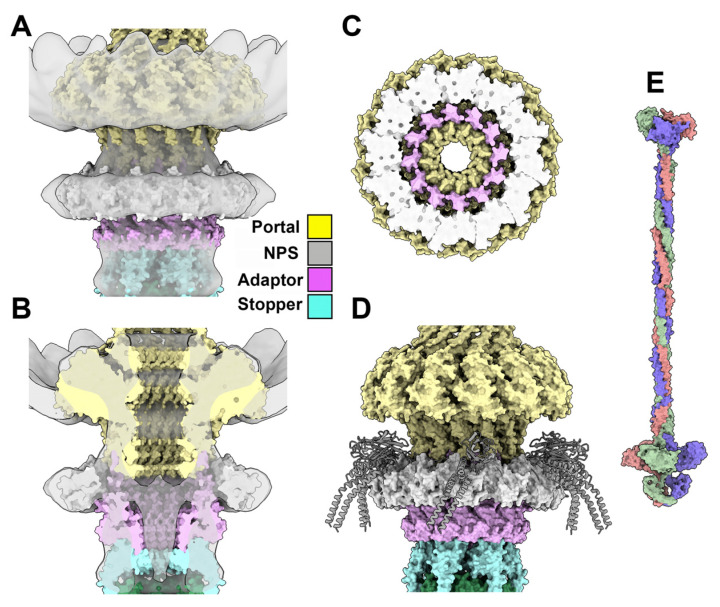
The fit of the neck in the nsEM density map. (**A**) The fit of the neck (portal, 12 mer, yellow; adaptor, 12 mer, pink; stopper, 6 mer, light blue; NPS N-termini, 12 mer, white) in the nsEM map at a 20.0 Å resolution. (**B**) Same view as in (**A**), but split at half its diameter. The NPS N-termini are located just below the portal/adaptor junction. (**C**) View at 90° of (**A**). The NPS N-termini cover the cavities observed at the portal/adaptor interface. (**D**) The phage 80α fiber structure was superimposed onto the NPS N-termini to illustrate a possible structure of the NPSs. (**E**) Surface view of the structural prediction of the NPS trimer with a total length of 48 nm.

While the overall fit is satisfying for the three modules (CC = 0.81), a ring-shaped density around the portal/adaptor interface cannot be interpreted with our model (Figure 6A,B). This density is positioned exactly on the “holes” of the adaptor surface. Interestingly, a similar ring-shaped density has previously been ascribed to a “collar” formed by the N-terminal domains of six neck passage proteins (NPSs) [47]. NPSs are elongated proteins, anchored to the neck, terminated by carbohydrate-binding modules. The NPSs of TP901-1 *(ORF51)* were first reported by Vegge et al. [47]. However, to our knowledge, nothing is known about the precise molecular attachment of these NPSs to the neck. The closest example of a structure resembling the collar is represented by the tail fibers FibU and FibL of the *Staphylococcus aureus* siphophage 80α [48], or the BppU N-terminal domain of phage TP901-1 [5]. In the *S. aureus* phage 80α, the six trimeric fibers (18 monomers) are attached to the phage tail by a ring of 12 N-terminal domains, plus six N-terminal domains located above the ring [48]. 

Our reasoning was that this arrangement might also apply to NPSs. AF2 predicted a ring of 12 N-terminal domains of NPSs (residues 1–125) that yielded excellent statistics and fitted well in the nsEM map (Figure 6a–c). We then attempted to predict a complex between the adaptor and 12 N-terminal domains of NPSs, which unfortunately failed. Nonetheless, fitting the FibU ring on top of the dodecameric NPS N-terminal domain ring might provide insights as to how the NPS is projected out of the tail axis (Figure 6D). 

### 3.4. The Neck/Tail Junction and the Tail

The structures of several protein complexes were predicted for the neck/tail junction and the tail: (i) the complex formed by the adaptor (minus its C-terminal helix), stopper, tail terminator (TT), and MTP (12/6/6/6 mer); (ii) the adaptor/stopper complex (12/6 mer); (iii) the stopper/TT complex (6/6 mer); (iv) the TT/MTP complex (6/6 mer); and (v) the MTP/MTP complex (6/6 mer).

#### 3.4.1. The Stopper/Tail Terminator (TT; ORF41) Complex

We predicted the structure of the complex between a stopper hexamer and a TT hexamer. The TT is composed of a six-stranded b-sheet (s1–s6) covered by two a-helices (h1–h2) inserted between b-strands s3 and s4 (Figure 4d). In the TT hexamer, the six b-sheets form a cylindrical b-barrel maintained by inter-monomer interactions between b-strands s2 and s5. The TT/TT interactions cover 840 Å^2^ of a total surface area of 7700 Å^2^ (~11%). A Dali search of the most closely related structures in the PDB reported a small number of relevant hits, among which the most pertinent was the retrieved structural similarity hit with the tail terminator of phage GTA (PDB ID: 6te9; Z-score = 14.0; rmsd = 2.4 Å; lali: 122/134; [43]).

In the stopper/TT complex, the stopper b-hairpins have a conformation different to that observed in the absence of the TT. While the stopper b-sandwich structure and the stopper/stopper interactions are maintained, the b-hairpins move by ~20° to embrace the TT hexameric ring (Supplementary Materials, Figure S4 and Supplementary Video S1). Each stopper b-hairpin stacks against the TT’s b-strand s5 of monomer i, and is sandwiched between the a-helix h2 of the monomer i and the a-helix h1 of the monomer i + 1. Additional contacts involve the TT’s long loops h1–s1 and s5–s6 and the stopper’s C-terminus and b-sandwich loops. Each stopper monomer interacts with two TT monomers and covers surface areas of 400 and 330 Å^2^, respectively, respectively (730 Å^2^ in total, ~10% of the total surface area). 

#### 3.4.2. The Tail Terminator (TT)/Major Tail Protein (MTP; ORF42) Complex

We predicted the structure of the complex between a TT hexamer and an MTP hexamer. The TT hexamer’s structure is similar to that observed in the absence of MTPs. The MTP structure displays a sandwich of two anti-parallel b-sheets (Figure 5E). The five b-strands of the external b-sheet show a connectivity between s1, s6, s7, and s10, and the four b-strands of the internal b-sheet show a connectivity between s2, s5, s9, and s8. An a-helix between b-strands s1 and s2 and a long b-hairpin between s3 and s4 complete the structure. The internal b-sheets in the MTP hexamer assemble as a tight anti-parallel b-barrel of 24 b-strands, maintained by interactions between b-strands s2 and s8. Additional inter-monomer contacts involve the N-terminus that sneaks into the adjacent monomer. The b-hairpin establishes strong contact with the adjacent MTP’s b-sandwich and b-hairpin. The a-helix is also in contact with the loops of the external b-sheet. The buried surface of these inter-MTP monomer interactions involves 1340 Å^2^ of the 12,200 Å^2^ total area (~11%). A Dali search identified several hits in the PDB, among which the MTP of phage SPP1 was the best (PDB ID: 6yeg; Z = 15.2; rmsd = 2.3 Å; lali: 157/172; [49]). The N-terminus of the MTP establishes contacts with adjacent MTP and TT moieties, while the C-terminus contacts the closest TT and the adjacent one (Figure 5E). The TT s2–s3 loop extends to contact the MTP’s internal b-sheet. Overall, each TT interacts with three MTPs: MTP i covers 770 Å^2^ of its surface, and MTPs i − 1 and i + 1 cover 380 Å^2^ and 110 Å^2^ of its surface, respectively, for a total surface area of 1260 Å^2^ (~16% of the TT surface).

#### 3.4.3. The MTP/MTP Rings

**Figure 7:**
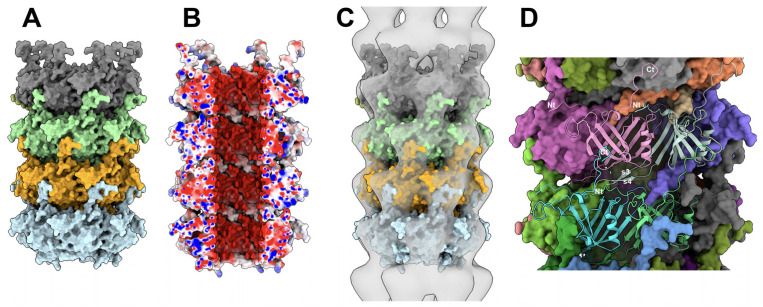
The tail tube. (**A**) Surface view of four MTP rings forming a section of the tail tube (the top of the view is the direction of capsid). Note the insertion of each monomer C-terminus inside a monomer cavity in the above ring. (**B**) Same orientation as in (**A**) but split at its half-diameter and colored according to electrostatics. (**C**) Fit of the MTPs in the nsEM map at 20Å resolution (EMD-2228). (**D**) MTP/MTP interactions between the stacked rings. Interactions involve the N- and C-termini and the b-hairpin with strands s3 and s4.

Employing AF2, we predicted three stacked rings, each consisting of MTP hexamers, representing a total of 3042 residues. We then added a fourth hexamer with Coot [35], leading to four stacked rings (Figure 7A). These four MTP rings fit into the 20.0 Å resolution nsEM 3D reconstruction (EMD-2228). However, the map had to be flipped to obtain the best result (CC = 0.79) (Figure 7B). The six MTP b-hairpins of a hexamer form a platform onto which the lower hexamer is stacked (Figure 7C). The contacts are established between the b-hairpin and the N-terminus of adjacent monomers, and between the C-terminus of a lower monomer and the external b-sheet of an upper monomer (Figure 7D). Each MTP of one ring contacts four MTPs of the other ring: the largest interaction surface is established with the nearest monomer i (835 Å^2^), and other interactions of 485, 105, and 45 Å^2^ are established with monomers i + 1, i + 2, and i − 1, respectively, with a total surface area of 1500 Å^2^ (~12.4% of the total surface).

### 3.5. The Baseplate

**Figure 8:**
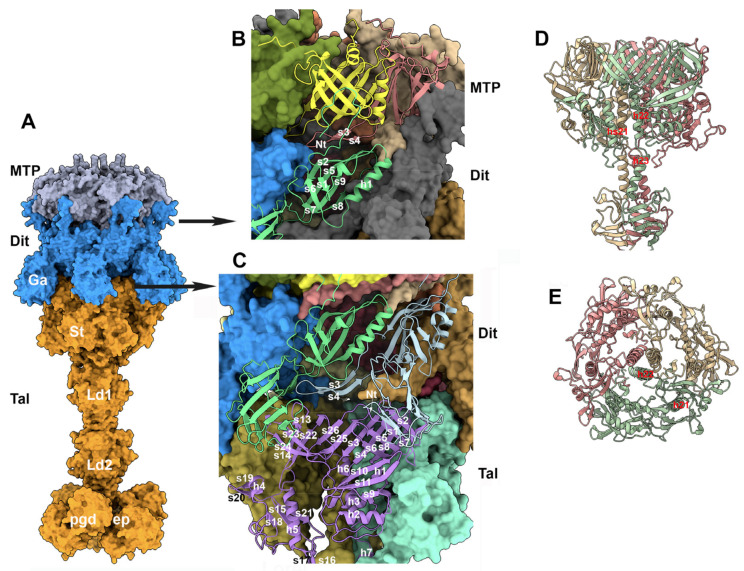
The distal MTP and the central baseplate. (**A**) Surface view of the distal hexameric MTP (gray), the hexameric Dit (blue), and the trimeric Tal (orange). Ga: the Dit’s galectin domain; St: the Tal’s conserved structural domain; Ld1 and Ld2: linker domains 1 and 2; pgd and endopeptidase (ep) domains. (**B**) Ribbon view of the MTP/Dit interface. (**C**) Ribbon view of the Dit/Tal interface. (**D**) Ribbon side-view of the Tal trimer N-terminus (residues 1–484). (**E**) Same view rotated by 90° within the phage’s main axis.

The baseplate of TP901-1 comprises four proteins: the Dit, Tal, BppU, and RBP (ORF46, 47, 48, and 49, respectively) [5,11,50,51]. The Dit hexamer and the Tal trimer form the central part of the baseplate and extend the last MTP ring out of the tail. Moreover, the tape measure protein (TMP; ORF45) has been proposed to fill the central channel of the tail, Dit, and Tal [26]. A crystal structure of the TP901-1 baseplate, without the Tal, has previously been reported [5]. In this complex, six BppU trimers are attached to the Dit hexamer, each trimer projecting three RBP trimers at the baseplate periphery. Our goal here was to assess the MTP/Dit interface and the Tal/Dit interface, and to model a complete baseplate structure. To this end, we predicted the structure of a complex of MTP (×6), Dit (×3), and Tal (×3) structural domains (residues 1–380) (3672 residues in total). We also predicted the structure of the full-length Tal trimer and superimposed it onto the MTP/Dit/Tal N-terminal predicted structure using Coot [35] (Figure 8A). Lastly, we predicted the structure of the trimeric BppU, the trimeric RBP, and their complexes, and compared them to the X-ray structures.

#### 3.5.1. The MTP/Dit Interface

The predicted structure of the Dit hexamer superimposes well onto the Dit crystal structure (rmsd = 0.93 Å) [5]. Each Dit monomer comprises an N-terminal domain (residues 1–145) forming a b-sandwich with an a-helix and a b-hairpin, and a C-terminal domain (residues 146–255) folded as a galectin-like b-sandwich, also found in other Dit proteins [25,52] (Figure 8B). The six N-terminal domains form the Dit central core, with a Dit/Dit interface burying a surface area of 1400 Å^2^, while the six C-terminal domains extend out of the ring and do not interact with each other. Each Dit monomer projects a b-hairpin (strands s3, s4) towards the neighboring monomer, thereby ensuring the cohesion of the hexamer, and forming a large part of the interface between the MTP and Dit rings. The Dit N-terminal segment (residues 1–12) extends towards the MTP, covering its external b-sheet (Figure 8B). Each MTP monomer contacts three Dit monomers, with buried surfaces of 910 Å^2^, 520 Å^2^, and 160 Å^2^ for monomers i, i + 1, and i + 2, respectively, accounting for a total surface area of 1590 Å^2^.

#### 3.5.2. The Trimeric Tal and the Dit/Tal Interface

The Dit/Tal interface has a six-fold to three-fold symmetry mismatch reported for all Dit/Tal structures in tailed phages [25,53,54]. Each of the two Tal sub-domains faces one Dit, thereby assembling a pseudo-six-fold symmetrical Tal ring (for details, see [53]). Two other domains form the lower part of the Tal, which were shown to undergo conformational changes when opening the Tal for dsDNA ejection [25].

Here, the TP901-1 Tal trimer is maintained in a semi-open position as it accommodates three h22 helices in its center (Figure 8D,E). They are followed by three h23 helices abutting the structural domains of three b-sheets made up of five anti-parallel b-strands each (Figure 8D). This b-domain is followed by a second b-stranded structural domain and the two enzymatic domains already described elsewhere [47,55]. The two Tal monomer domains in contact with the Dit form a b-sandwich, the internal part being formed by eight b-strands. The Tal trimer is therefore tightly assembled through a 24 b-strand continuous belt (Figure 8C,D). The two Tal monomer (i) domains located below the belt do not interact with one other, each interacting with the i − 1 and i + 1 Tal monomer (Figure 8C). The interactions between the Dit ring and the Tal trimer are again mediated by a b-hairpin of the Dit (s3–s4), but also by the interactions of the Dit’s galectin domains with the two upper domains of the Tal (Figure 8A,C). Each Tal interacts with three Dit monomers: a major interaction with a Dit (1240 Å^2^) and two other interactions with flanking molecules of 360 Å^2^ and 510 Å^2^, respectively, with a total surface area of 2110 Å^2^, thus accounting for 10% of the total Tal surface area. 

#### 3.5.3. The Dit/BppU and BppU/RBP Complexes

**Figure 9:**
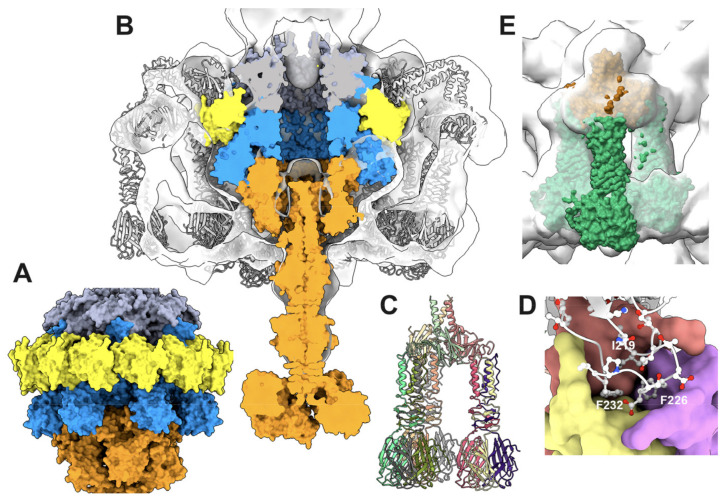
The peripheral baseplate. (**A**) Surface view of the dodecameric BppU N-terminal domain (yellow) complexed to the hexameric Dit (blue): MTP: gray; Tal: orange. (**B**) Split view of the nsEM map (EMD-1793; 25 Å resolution) of the complete baseplate with the distal hexameric MTP (gray), the hexameric Dit (blue), the trimeric Tal (orange), and the dodecameric BppU N-terminal domain (yellow) fitted inside. The gray ribbon inside the map represents the X-ray structure (PDB id 4v96). (**C**) The AF2-predicted structure of the “tripod”, formed of the trimeric BppU C-terminal domain holding three RBP trimers. (**D**) Close-up of the BppU/RBP interface with the hydrophobic residues Ile219, Phe226, and Phe232 also identified in the X-ray structure. (**E**) Surface view of the “tripod” fit in the nsEM map.

The structure of the hexameric Dit with the 18 BppU monomers is currently beyond the capacity of AF2 prediction. We therefore tried different predictions involving the hexameric Dit and three or six BppU monomers, without success. However, like for the NPS–neck complex (see above), we were able to successfully predict the structure of the hexameric Dit with a dodecamer of the BppU N-terminal domain (residues 1–140) (Figure 9A,B) that superimposes well onto the TP901-1 baseplate’s crystal structure. 

We also successfully retrieved the predicted structure of three BppU with three RBP trimers. While the structure of the N-terminus (1–170) of BppU is not assembled as it is in the X-ray structure, due to the absence of the Dit hexamer, the following helical domain and C-terminal RBP attachment domains are folded as in the X-ray structure. Furthermore, the attachment of the RBP trimer to its BppU receptor is very close to that observed in the X-ray structure (PDB: 4v96) (Figure 9C). The BppU/RBP complex fits well in the nsEM map (EMD-1793) (Figure 9E). Even at the side-chain level, three BppU hydrophobic residues (Ile219, Phe226, and Phe232) that were shown in the X-ray structure to play a crucial role in the BppU/RBP interaction are observed in the same conformations [5] (Figure 9D). Finally, the interactions of the TMP within the virion could not be deciphered. The TMP was shown to participate in the initial step of the tail tube formation by assembling to the Dit/Tal/BppU/RBP complex and being assisted by specific chaperones [23]. Moreover, it was shown that the TMP forms a trimer within the phage tail tube, down to the Tal trimer [10,48,56], indicating that the TMP should interact with the TT, MTP, and Dit hexamers, as well as with the Tal trimer. We therefore tried to predict the interactions of: (i) a trimer of the TMP N-terminus with a TT hexamer; (ii) a trimer of the TMP middle region with an MTP hexamer; and (iii) a trimer of the TMP C-terminus with the Dit hexamer/Tal trimer, all without any success. In all cases, a trimer of the TMP could not be predicted. This may be due to the fact that the TMP assembly is driven by chaperones that form transient structural states, as reported previously [23].

## 4. Discussion

In our predicted structure of the TP901-1 virion, the hexons and pentons of the capsid were well predicted, except for the N-termini, as they may be involved in interactions with other hexons or pentons. Our predictions of hexon/hexon or hexon/penton interactions were unsuccessful: the hexons and penton were predicted as separate units without any interactions. Hence, AF2 cannot determine the contents of an icosahedral asymmetric unit and cannot predict the T-number nor handedness.

The procapsid assembly requires a so-called scaffolding protein (SP), which acts as a chaperone to ensure the correct positioning of the subunits [57,58]. The structures of full or partial SPs have been previously reported (PDB ID: 1tx9, 2gp8, 1no4, 8dt0); they are helical proteins, as seen in the phage Phi29, where they form a dimer of two long a-helices [57]. In the procapsid of staphylococcal phage 80α, the SP C-terminal a-helix was observed to be in contact with each of the capsid components [59]. In phage TP901-1, the predicted structure of the expression product of ORF35, which is located immediately upstream of the MCP-encoding gene, displays a pattern of two long helices and some shorter ones, making it a good candidate for being an SP.

The predicted dodecameric portal exhibits all the characteristics of genuine phage portals previously reported in the literature [45,60,61]. However, the TP901-1 portal has a unique feature: its clip domain contains three β-strands originating from two different portals, and a fourth β-strand coming from the adaptor. The TP901-1 adaptor also presents a unique feature, with a 24-stranded β-barrel forming its internal channel. Interestingly, the not-compact and rather loose interface between the portal and adaptor is compensated by the presence of a ring of six NPS trimers, whose N-terminal domains cover the adaptor ring. The hexameric stopper of TP901-1 is similar to that of other phages and exhibits long b-hairpins that embrace the tail tube upon complexation.

The tail and baseplate components, including the TT, MTP, and Dit proteins, share structural features that have previously been reported [15,62]. A superimposition of their monomers reveals that these three components possess a β-hairpin that interacts with the other subunits of a hexameric ring and also provides a stacking platform between the hexameric rings. The MTP and Dit proteins also possess an extended N-terminus that inserts into a crevice of an above hexamer in the MTP/MTP and Dit/MTP interactions. While platforms of β-hairpins were also observed in the MTP stacking of the tailed phages 80α [48] and T5 [56,63], the N-terminal lock was only observed in 80α, for which a β-helix helps the interaction.

The structure of a TP901-1 baseplate has previously been reported, though without its Tal component [5]. Here, the full-length Tal completes the X-ray structure. The central channel of the Tal N-terminal structural domain (1–380) is filled by three α-helices that link this domain to the functional C-terminal domain. Surprisingly, this central channel is filled in phage 80α by the C-terminus of the three TMPs contained in the tail tube [48]. However, the position of the three helices in TP901-1 Tal is logical considering the Tal topology. Indeed, these helices and the C-terminal domain should dramatically rearrange to allow the TMP to exit and for DNA ejection during the initial stages of infection. Such a rearrangement has recently been reported for phage T5. Upon contact of the tail tip with T5′s receptor (the membrane protein FhuA), the Tal-like protein pb3, which obstructs the tail exit channel, opens and rotates on the tail side, thus allowing the TMP (pb2) to insert into the membrane [10,56]. We postulate that a similar mechanism is operating in the case of phage TP901-1 upon its baseplate/cell wall polysaccharide binding. 

Our prediction of the RBP structures and their interaction with BppU was excellent, as was the prediction and localization of 12 out of the 18 BppU N-terminal domains that form a ring similar to the NPS ring or to the tail fiber rings observed in phage 80α [48]. However, the six remaining BppU N-terminal domains and the BppU topology were out of reach due to the fact that the complete structure is well over AF2’s residue limit. Lastly, all attempts to model the TMP in complex with the MTP, Dit, and tail chaperone were unsuccessful.

To conclude, we have found that AF2 has impressive prediction capabilities, provided that the ensemble used for prediction involves a residue limit of less than around 4000. Most of our prediction failures were primarily linked to this limitation. However, as evidenced by the capsid and parts of the baseplate, the limitations of this study may not be due to this factor only. Despite this, we illustrate here that AF2’s predictions provide an avenue for further investigation into phage structures. This is particularly important for phages that remain poorly characterized at the structural level, such as those infecting human pathogens and mycobacteria [64].

## Data Availability

Coordinates of predicted structures are accessible on Zenodo (https://doi.org/10.5281/zenodo.10372858).

## Supplementary Materials

The following supporting information can be downloaded at https://www.mdpi.com/article/10.3390/v15122440/s1: Figure S1: AF2-predicted local distance difference test (pLDDT) and predicted aligned errors (PAEs) of the complexes between TP901-1 components. Figure S2: AF2-predicted structures of the capsid’s hexon and penton and the portal dodecamer colored according to the pLDDT values (from low, blue to high, red). Figure S3: Ribbon view of the structures of two hexons (h1 and h2) and one penton (P) fitted in the nsEM 3D reconstruction (EMD-2133). Figure S4: The capsid–neck assembly with the tail–baseplate. Video S1: ChimeraX [33] morphing of the stopper (top)/tail–terminator (bottom) docking. Representation of atoms as spheres.
