# Supplementary material for: Partial Atomic Model of the Tailed Lactococcal Phage TP901-1 as Predicted by AlphaFold2: Revelations and Limitations
Source: Viruses. 2023 Dec 15;15(12):2440. doi: 10.3390/v15122440 (PMC10747895; doi:10.3390/v15122440)
Supplement: Supplementary file 1 [file viruses-15-02440-s001.zip › viruses-2740258-supplementary.pdf]

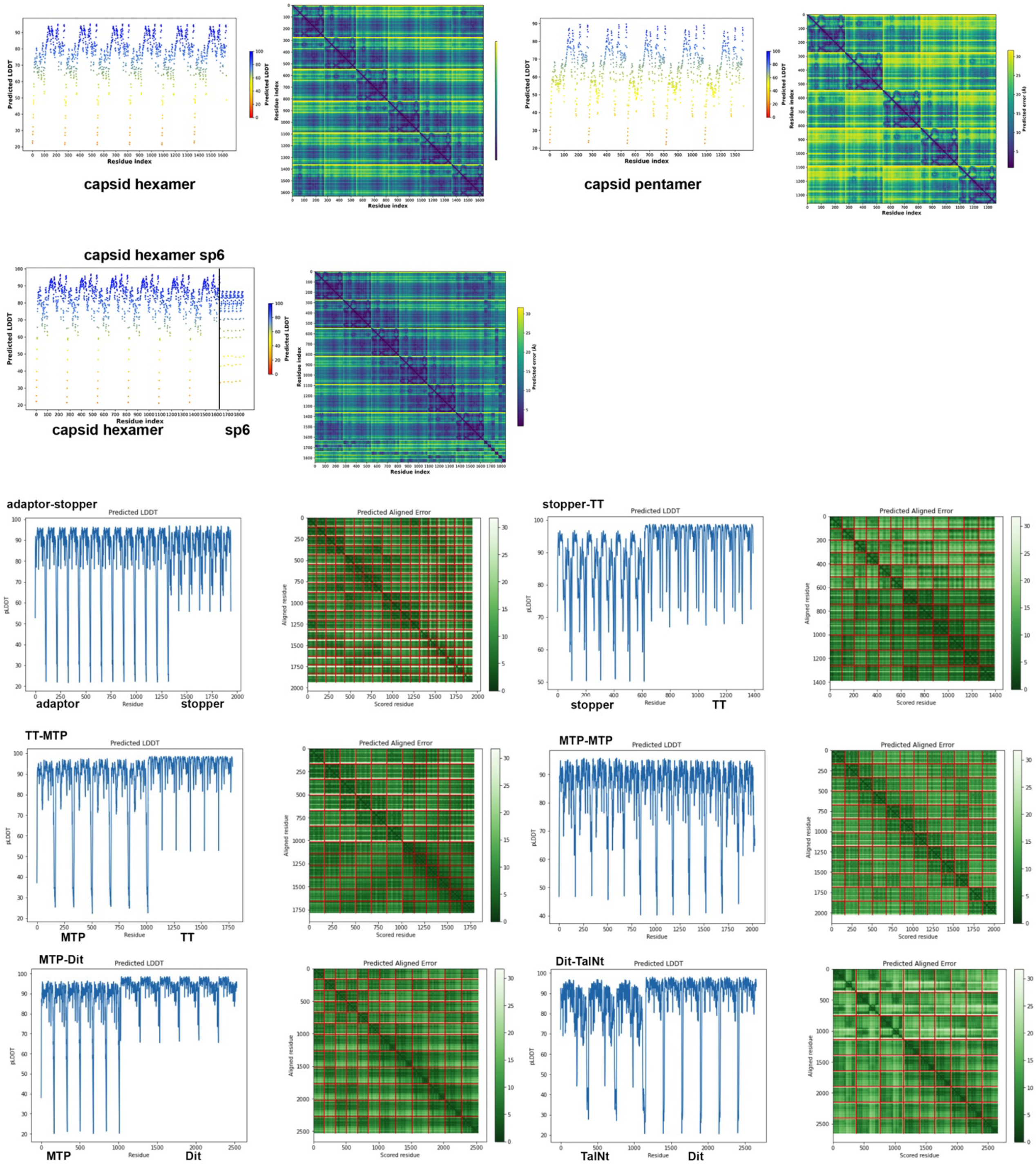

Supplementary Figure S1: AlphaFold predicted local distance difference test (pLDDT) and predicted aligned errors (PAE) of the complexes between TP901-1 components.

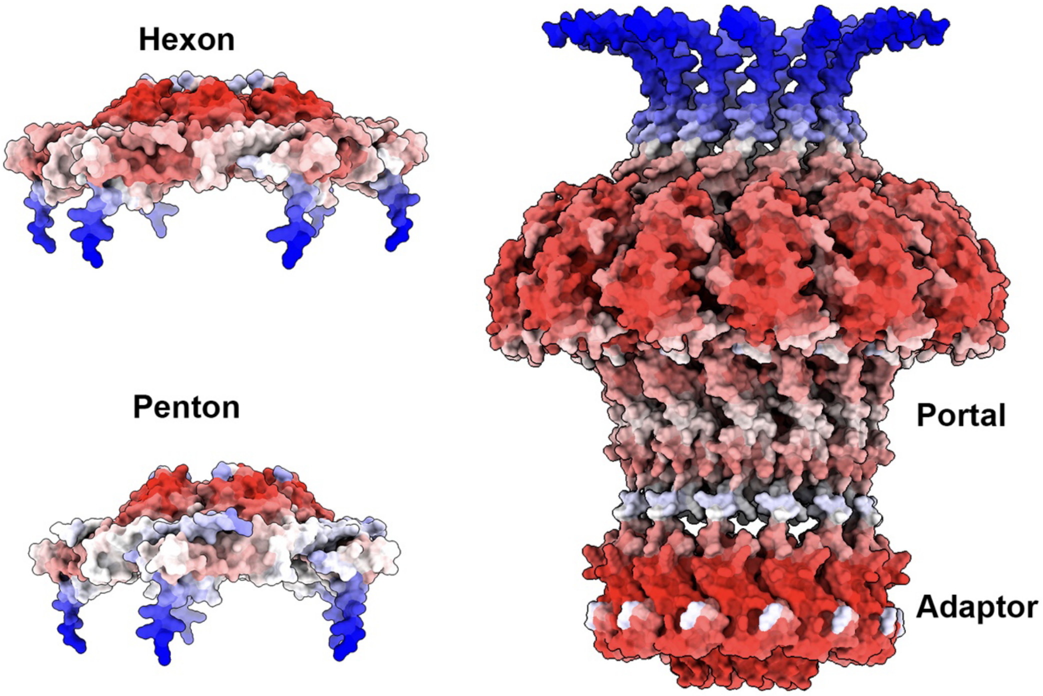

Supplementary Figure S2: AlphaFold predicted structures of the capsid's hexon and penton and the portal dodecamer colored according to the pLDDT values (from low , blue to high, red).

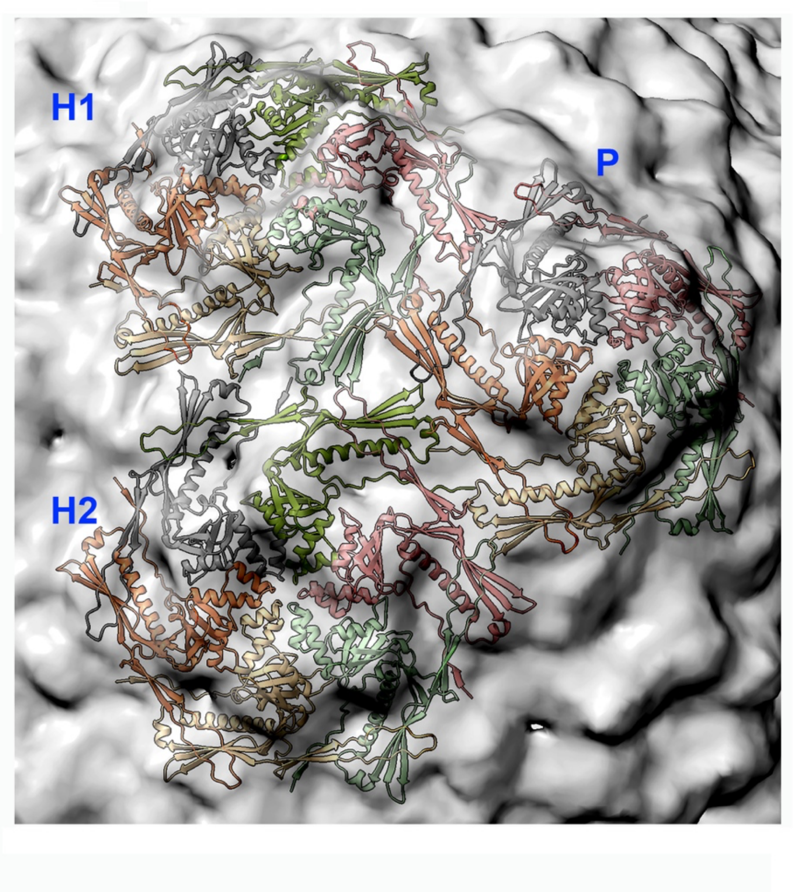

Supplementary Figure S3: Ribbon view of the structures of two hexons (H1 and H2) and one penton (P) fitted in the nsEM 3D reconstruction (EMD-2133).

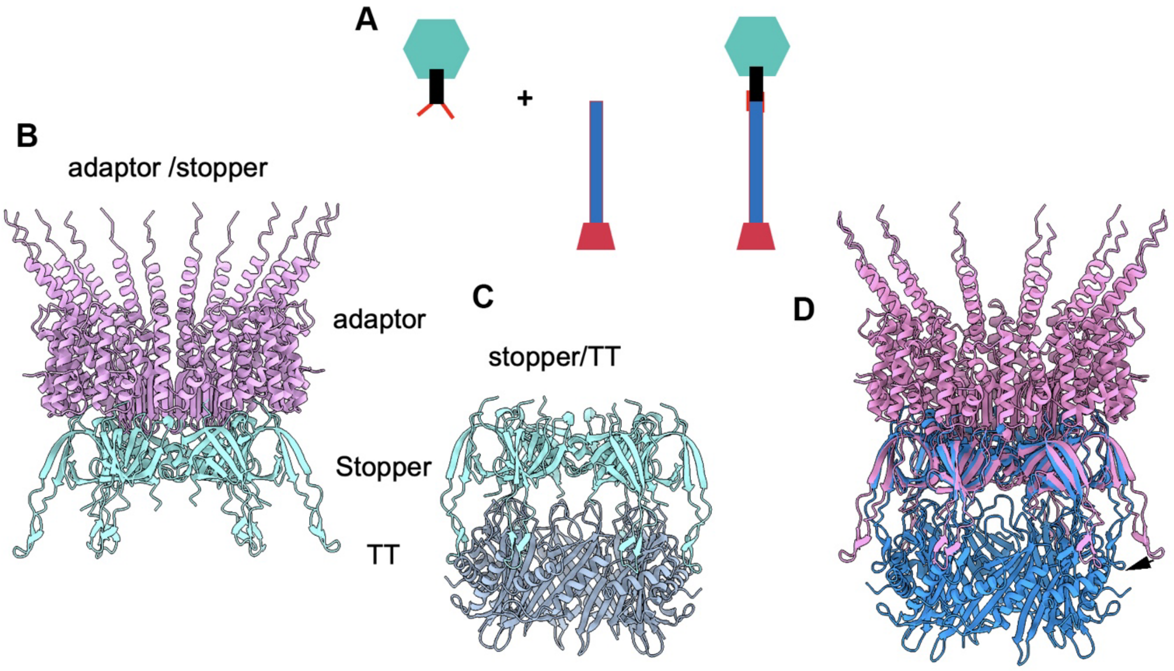

Supplementary Figure S4: **The capsid-neck assembly with the tail-baseplate.** (**A**) Schematic representation of the last step of a siphophage assembly. The capsid-neck and the tail-baseplate ensembles are assembled separately and joined together in a last step through a stopper/tail-terminator contact. (**B**) Ribbon view of the dodecameric adaptor (pink) and the hexameric stopper (blue) complex. (**C**) Ribbon view of the hexameric stopper (blue) and the hexameric tail-terminator (TT; grey) complex. (**D**) Superposition of the ribbon view of the dodecameric adaptor and the hexameric stopper complex (blue) on the hexameric stopper and the hexameric tail-terminator (TT) complex (blue). Note hox the b-hairpins of the stopper rotate (by 20°) to contact the TT (black arow on the left side).

Supplementary Video S1: ChimeraX [33] morphing of the stopper (top) / tail-terminator (bottom) docking. Representation of atoms as spheres.

[33] Pettersen, E. F., T. D. Goddard, C. C. Huang, G. S. Couch, D. M. Greenblatt, E. C. Meng, and T. E. Ferrin. "Ucsf Chimera--a Visualization System for Exploratory Research and Analysis." *J Comput Chem* 25, no. 13 (2004): 1605-12.
